# Supplementary material for: Workplace Mistreatment and Health Conditions Prior and during the COVID-19 in South Korea: A Cross-Sectional Study
Source: Int J Environ Res Public Health. 2022 Oct 11;19(20):12992. doi: 10.3390/ijerph192012992 (PMC9603205; doi:10.3390/ijerph192012992)
Supplement: Supplementary file 1 [file ijerph-19-12992-s001.zip › ijerph-1904138-supplementary.pdf]

Supplementary Table S1. Results of factors associated with having health problems in 2017 and 2020–2021 by models.

Mistreatment type	Men								Women							
	Headaches		Anxiety		Overall fatigue		Physical pain		Headaches		Anxiety		Overall fatigue		Physical pain	
**Model 1***	**OR**	**95% CI**	**OR**	**95% CI**	**OR**	**95% CI**	**OR**	**95% CI**	**OR**	**95% CI**	**OR**	**95% CI**	**OR**	**95% CI**	**OR**	**95% CI**
**Discrimination**																
No	1.00		1.00		1.00		1.00		1.00		1.00		1.00		1.00	
Yes	2.20	(1.99 - 2.45)	2.97	(2.21 - 4.00)	1.31	(1.15 - 1.50)	0.87	(0.74 - 1.02)	2.41	(2.19 - 2.65)	2.37	(1.73 - 3.23)	1.20	(1.06 - 1.36)	1.01	(0.89 - 1.14)
**Abuse**																
No	1.00		1.00		1.00		1.00		1.00		1.00		1.00		1.00	
Yes	3.52	(2.46 - 5.05)	9.60	(5.22 - 17.64)	1.39	(0.85 - 2.28)	1.11	(0.63 - 1.93)	2.98	(2.36 - 3.76)	4.25	(2.40 - 7.52)	1.23	(0.89 - 1.68)	1.20	(0.88 - 1.63)
**Overworking**																
No	1.00		1.00		1.00		1.00		1.00		1.00		1.00		1.00	
Yes	2.39	(2.15 - 2.66)	2.93	(2.16 - 3.98)	1.34	(1.17 - 1.53)	1.07	(0.91 - 1.24)	2.17	(1.85 - 2.54)	3.07	(1.99 - 4.75)	1.16	(0.95 - 1.43)	1.11	(0.91 - 1.36)
**Model 2†**	**OR**	**95% CI**	**OR**	**95% CI**	**OR**	**95% CI**	**OR**	**95% CI**	**OR**	**95% CI**	**OR**	**95% CI**	**OR**	**95% CI**	**OR**	**95% CI**
**Discrimination**																
No	1.00		1.00		1.00		1.00		1.00		1.00		1.00		1.00	
Yes	2.49	(2.24 - 2.77)	2.95	(2.18 - 3.98)	1.31	(1.14 - 1.49)	0.95	(0.81 - 1.11)	2.62	(2.38 - 2.89)	2.40	(1.76 - 3.27)	1.27	(1.12 - 1.44)	1.15	(1.01 - 1.30)
**Abuse**																
No	1.00		1.00		1.00		1.00		1.00		1.00		1.00		1.00	
Yes	3.48	(2.43 - 5.00)	9.59	(5.23 - 17.58)	1.43	(0.87 - 2.34)	1.11	(0.63 - 1.99)	2.99	(2.38 - 3.77)	4.32	(2.44 - 7.62)	1.24	(0.90 - 1.71)	1.18	(0.86 - 1.62)
**Overworking**																
No	1.00		1.00		1.00		1.00		1.00		1.00		1.00		1.00	
Yes	2.55	(2.29 - 2.84)	2.89	(2.12 - 3.93)	1.30	(1.13 - 1.49)	1.09	(0.93 - 1.27)	2.30	(1.97 - 2.69)	3.10	(2.00 - 4.81)	1.23	(1.00 - 1.51)	1.26	(1.03 - 1.56)
**Model 3‡**	**OR**	**95% CI**	**OR**	**95% CI**	**OR**	**95% CI**	**OR**	**95% CI**	**OR**	**95% CI**	**OR**	**95% CI**	**OR**	**95% CI**	**OR**	**95% CI**
**Discrimination**																
No	1.00		1.00		1.00		1.00		1.00		1.00		1.00		1.00	
Yes	2.40	(2.16 - 2.67)	2.83	(2.09 - 3.83)	1.29	(1.13 - 1.48)	0.95	(0.81 - 1.11)	2.51	(2.27 - 2.77)	2.38	(1.74 - 3.26)	1.30	(1.14 - 1.47)	1.20	(1.05 - 1.36)
**Abuse**																
No	1.00		1.00		1.00		1.00		1.00		1.00		1.00		1.00	
Yes	3.38	(2.35 - 4.85)	8.28	(4.50 - 15.23)	1.26	(0.76 - 2.08)	1.01	(0.56 - 1.83)	3.06	(2.42 - 3.85)	4.29	(2.42 - 7.60)	1.18	(0.86 - 1.64)	1.11	(0.81 - 1.53)
**Overworking**																
No	1.00		1.00		1.00		1.00		1.00		1.00		1.00		1.00	
Yes	2.40	(2.15 - 2.68)	2.45	(1.77 - 3.37)	1.10	(0.95 - 1.27)	0.98	(0.83 - 1.15)	2.15	(1.83 - 2.52)	3.02	(1.96 - 4.65)	1.17	(0.95 - 1.45)	1.29	(1.05 - 1.60)

Note: total N. of participants – 44 425 (all participants), *not adjusted, †adjusted to age and year, ‡ adjusted to age, year, educational level, income amount/month, working hours/week.
